# Development and application of a scale for older patients’ perceived ageist behavior of medical staff in China

**DOI:** 10.3389/fpsyg.2026.1791211

**Published:** 2026-04-14

**Authors:** Jingpin Ren, Yue Wang, Lurong Liu

**Affiliations:** 1 School of Management, Chengdu University of Traditional Chinese Medicine, Chengdu, China; 2 Deyang Hospital Affiliated Hospital of Chengdu University of Traditional Chinese Medicine, Deyang, China; 3 School of Basic Medical Sciences, Chengdu University of Traditional Chinese Medicine, Chengdu, China

**Keywords:** ageism, ageist behavior, medical staff, older patients, patient loyalty

## Abstract

**Objective:**

To develop and validate the Older Patients’ Perceived Ageist Behavior of Medical Staff (OPABMS) Scale, assess the current status of OPABMS, and explore the association between OPABMS and older patients’ loyalty (OPL), thereby providing an evidence base for reducing ageism and improving patient retention.

**Methods:**

The OPABMS scale was developed through a multi-stage process involving literature review, expert consultation, in-depth interviews, and pilot survey, after which item analysis, exploratory factor analysis, confirmatory factor analysis, convergent validity, discriminant validity, criterion validity, reliability analysis, measurement invariance, and common method bias testing were performed to evaluate its psychometric properties. A total of 1,010 older patients who had visited medical institutions in the past year were recruited as a convenience sample. Data were collected using a sociodemographic questionnaire, the OPABMS Scale, the Patient Loyalty Scale and the Patient Satisfaction Scale.

**Results:**

The scale comprises 13 items grouped into three dimensions: Avoidance Behavior (AB), Perfunctory Behaviors (PB) and Complaint Behavior (CB). The overall mean score for OPABMS was 2.08 (*SD* = 0.65), with AB scoring highest, followed by PB and CB. The mean score for OPL was 3.45 (*SD* = 0.77). Significant differences in OPABMS were observed across age, usual residence, occupation, education, and household income (*p* < 0.05). Total and subscale scores of OPABMS were negatively correlated with OPL scores (*r* = −0.390 to −0.240, *p* < 0.001). Hierarchical regression showed that AB accounted for 11.6% of the variance in Intention to Recommend; AB and PB together explained 8.4% of the variance in Intention to Revisit and 14.1% in Intention to Spread Positive Word of Mouth.

**Conclusion:**

The OPABMS Scale demonstrates satisfactory reliability and validity and can thus serve as an appropriate instrument for measuring OPABMS. Older patients perceived a slightly below-moderate level of ageist behaviors from medical staff. These perceived behaviors were negatively associated with OPL. Health authorities, medical institutions, and medical staff should pay greater attention to OPABMS and its potential negative implications.

## 1 Introduction

The accelerating pace of global aging has become a significant public health issue (Shen et al., 2021), and China is one of the countries most severely affected by population aging (Fang et al., 2020). Data indicate that by the end of 2024, the proportion of China’s population aged 60 and above had reached 23.0%, formally marking the country’ s entry into a moderately aging society (National Bureau of Statistics of China, 2026).

Ageism was first introduced by Butler (1969), the president of the International Longevity Center-USA, who defined it as individual and societal prejudices and discriminatory attitudes toward older persons and the elderly as a group. Initially, ageism was predominantly conceptualized as a negative attitude toward older adults; as research has gradually advanced, the connotation of ageism has grown substantially more nuanced and multifaceted. Traxler (1980) reconceptualized ageism as “any attitude, action, or institutional structure that judges individuals or groups on the basis of age, or assigns social roles purely according to age,” thereby encompassing not only attitudinal bias but also behavioral and institutional ageism. While the World Health Organization is leading the global fight against ageism (Browning et al., 2025), numerous studies have documented its pervasive presence in healthcare settings (Dix et al., 2024; Burnes et al., 2019; Van Wicklin, 2020), such as medical staff attaching lower priority to older patients (Skirbekk and Nortvedt, 2014) and older adults consistently being assigned the lowest treatment priority (Rosenbaum, 2020). Research indicates that ageist behaviors of medical staff significantly undermine the physical and mental health of older patients (Hu et al., 2021; Chang et al., 2020; Allen et al., 2022), underscoring the need for urgent attention.

Older patients’ perceived ageist behavior of medical staff (OPABMS) refers to the subjective experience of older patients perceiving differential or unfair negative or harmful treatment from medical staff. It represents a subjective, negative healthcare experience, rather than an objective evaluation of clinical quality or professional behavior. Within healthcare settings, ageist behaviors of medical staff not only diminish older patients’ healthcare experience but also compromise treatment effectiveness (Grant et al., 2000; Peake et al., 2003). Social Information Processing Theory was first proposed by Salancik and Pfeffer (1978). The theory holds that an individual’s attitudes and behaviors are influenced by information from the surrounding environment; external social information interacts with individual characteristics to shape attitudes such as satisfaction, which in turn guide behavioral choices (Salancik and Pfeffer, 1978; Montani et al., 2020). Concurrently empirical work has demonstrated that a positive care experience is a critical prerequisite for enhancing patient loyalty (Arslan et al., 2022). Therefore, combining the theory with previous research, we infer that OPABMS, as a negative care experience, is negatively associated with older patients’ loyalty (OPL) to the medical institution, thereby hindering both the quality management of geriatric health services and the construction of age-friendly healthcare facilities. Social Information Processing Theory serves as the theoretical foundation for clarifying the current status of OPABMS and its association with OPL.

Existing research, both domestically and internationally, has largely focused on ageist attitudes, with relatively limited attention devoted to the study of ageist behaviors. Most current assessment tools evaluate the level of ageism among perpetrators based on their attitudes, with the Relating to Older People Evaluation (ROPE) (Cherry and Palmore, 2008) questionnaire being the only instrument that assesses ageism through the lens of behaviors. Compared to ageist attitudes, ageist behaviors can be more directly perceived by the targets of ageism and have a more immediate impact on them. Besides, previous studies have primarily adopted a single perspective, often relying on self-assessment methods to evaluate the level of ageism among perpetrators. To date, little research has been reported that assesses ageist behaviors of perpetrators from the perspective of the targets, namely older adults. As the direct recipients of ageist behaviors, older patients are better positioned to provide a more authentic reflection of such behaviors, as targets of ageism tend to be more sensitive and are more likely to detect subtle forms of ageism (Abrams et al., 2016). Currently, research on ageism in the healthcare sector in China remains in its early stages; studies focusing on OPABMS have yet to be reported, and no corresponding measurement instrument is available.

Accordingly, this study aims to (a) develop the OPABMS Scale, (b) describe the current status of such perceptions, and (c) explore the association between OPABMS and OPL, thereby providing a theoretical basis for reducing ageism, improving older patients’ healthcare experiences, and enhancing their loyalty to medical institutions.

## 2 Materials and methods

### 2.1 Participants

We recruited older patients who had visited a medical institution within the past year. Due to the sensitivity and potentially accusatory nature of OPABMS, older patients may exhibit courtesy bias when evaluating such behaviors in healthcare settings, meaning they may refrain from or be unwilling to respond honestly in order to ingratiate themselves with medical staff. Therefore, to minimize survey bias, alleviate older patients’ psychological concerns, enable them to report their genuine healthcare experiences, and ensure the authenticity and reliability of the data, we did not conduct the survey in healthcare settings; instead, it selected only those older patients who had visited a medical institution within the past year. Inclusion criteria were: (a) aged ≥60 years; (b) at least one healthcare visit in the previous 12 months; (c) mentally competent and able to communicate verbally; (d) informed consent to participate.

Participants were selected by convenience sampling. Before administration, interviewers explained the purpose and procedure, obtained informed consent, and assured confidentiality to minimise reluctance. For respondents with limited literacy or poor eyesight, the interviewer read each item aloud and recorded the answers provided. We conducted two waves of data collection. In the first wave, 300 valid questionnaires (Sample 1) were obtained and used for the initial item analysis and exploratory factor analysis (EFA). In the second wave, 710 valid questionnaires were collected; this sample was randomly split into two halves: one half (Sample 2, *n* = 355) was used for the second item analysis and EFA, and the other half (Sample 3, *n* = 355) was used for confirmatory factor analysis (CFA).

### 2.2 Measures

#### 2.2.1 Development and validation of OPABMS scale

First, we conducted a comprehensive review of the domestic and international literature on ageism, perceived ageism, and ageist behaviors in medical institutions. By synthesizing existing definitions, dimensional frameworks, and measurement tools, we developed an operational definition of OPABMS. On this basis, and with input from four experts in gerontology and healthcare quality, we drafted an interview guide. Second, in-depth interviews were conducted with 23 older patients; the transcripts were imported into QSR NVivo 14.0 and analyzed sentence-by-sentence following grounded-theory procedures. Behaviors mentioned by at least three participants were retained, yielding 38 initial items. Third, older adults were asked to evaluate the clarity and acceptability of each item, and domain experts were invited to conduct a formal evaluation and provide critical feedback. Following iterative discussion and revision, a preliminary scale consisting of 32 items was formulated. The scale employs a 5-point Likert scoring system ranging from 1 (Never) to 5 (Always). Higher total scores indicate a greater degree of perceived ageist behavior by medical staff from the perspective of older patients.

#### 2.2.2 Patient loyalty scale

The Patient Loyalty Scale developed by Choi et al. (2004) was adopted. It comprises three dimensions [Intention to Recommend (I-REC), Intention to Revisit (I-REV), and Intention to Spread Positive Word of Mouth (I-PWM)], and each scored on a 5-point Likert scale ranging from 1 (strongly disagree) to 5 (strongly agree). Higher scores indicate greater patient loyalty to the medical institution. In this study, the Cronbach’s alpha value of the Patient Loyalty Scale was 0.823, indicating satisfactory reliability.

#### 2.2.3 Patient satisfaction scale

The Patient Satisfaction Scale translated and revised by Chen (2009) was adopted. It includes 5 items and uses a 5-point Likert scoring method. Higher scores indicate higher patient satisfaction with the medical institution. In this study, the Cronbach’s alpha value of the Patient Satisfaction Scale was 0.836, indicating satisfactory reliability.

### 2.3 Procedure

All participants were invited to complete measures in the following order: (a) demographic information: gender, age, usual residence, marital status, occupation, educational level, presence of accompanying family members during routine medical visits, and average monthly household income per capita (CNY); (b) OPABMS Scale; (c) Patient Loyalty Scale; (d) Patient Satisfaction Scale.

### 2.4 Data analysis

Data were managed and analyzed with SPSS 26.0, AMOS 28.0 and Mplus 8. (a) Item analysis: Pearson correlation and critical-ratio methods were employed. Items were retained if their correlations were significant and ≥0.40, indicating satisfactory discrimination (Sun et al., 2022). For the critical-ratio method, the total scale score was ranked from high to low; the top 27% of cases formed the high-score group and the bottom 27% the low-score group. Independent-samples t-tests compared the two groups on each item, items with statistically significant differences (*p* < 0.05) were retained as having good discrimination, while non-significant items were deleted (Kong and Lu, 2024). (b) Validity analysis: construct validity was used to evaluate the validity of the scale. Construct validity was examined through exploratory factor analysis (EFA) and confirmatory factor analysis (CFA). EFA began with Kaiser-Meyer-Olkin (KMO) and Bartlett’s tests: KMO > 0.800 and a significant *χ*^2^ (*p* < 0.05) indicated suitability for factorisation (Kaiser, 1974). The principal component analysis method with varimax rotation extracted factors with eigenvalues >1; a cumulative variance ≥50% was considered acceptable (Chen et al., 2021). CFA was performed with AMOS 24.0: the extracted factors were modelled in a structural equation framework and evaluated with *χ*^2^/df, GFI, AGFI, CFI, NFI, RMSEA, etc. (Xiang et al., 2022). We also calculated the Average Variance Extracted (AVE) and Composite Reliability (CR) values to assess convergent validity, and the Heterotrait-Monotrait Ratio (HTMT) to assess discriminant validity. To evaluate criterion validity, patient satisfaction was used as the criterion, and Pearson correlation analysis was conducted between patient satisfaction and the various dimensions of the OPABMS. (c) Reliability analysis: Internal consistency (the Cronbach’s Alpha value) and split-half reliability were computed, and the Cronbach’s Alpha value >0.70 for both were taken as evidence of good reliability (Youssef et al., 2023). (d) Measurement invariance: To test model fit across usual residence and gender, we tested four levels of measurement invariance: configural, metric, scalar, and strict invariance. Changes in CFI and RMSEA were considered acceptable if ≤0.01 (Cheung and Rensvold, 2002). (e) Common method biases test: Common method bias was assessed using the unmeasured latent method construct (ULMC) technique. Changes in CFI and TLI < 0.01, and changes in RMSEA and SRMR <0.05, indicate no serious common method bias (Wen et al., 2023; Lei et al., 2023).

Continuous variables are presented as *M(SD)*, and categorical variables as frequency and percentage (%). Group differences in OPABMS scores were examined using independent-samples t-test and one-way ANOVA. Pearson correlation analysis was employed to assess the relationship between OPABMS and OPL. Multicollinearity was assessed using the variance inflation factor (VIF) before conducting the hierarchical regression analysis. VIF > 5 or tolerance <0.2 indicated the existence of multiple collinearities (Qiu et al., 2023). Hierarchical regression analysis was used to explore the association between OPABMS and OPL. Statistical significance was set at *p* < 0.05.

## 3 Results

### 3.1 Psychometric properties of the OPABMS scale

#### 3.1.1 Exploratory factor analysis of the OPABMS scale

We conducted the first item analysis and EFA on Sample 1. Items were eliminated according to the following criteria: (a) communality <0.20; (b) the highest factor loading <0.40; (c) the difference between the two highest cross-loadings <0.20; (d) any factor containing fewer than three items (Leng et al., 2025; Akkaraputtapong et al., 2025). After deleting 9 items, the remaining 23 items were subjected to a second item analysis and EFA on Sample 2.

The second item analysis: correlation analysis showed that item–total correlations ranged from 0.528 to 0.735 (*p* < 0.001), indicating satisfactory homogeneity. Critical-ratio tests yielded *t* values of 10.634–17.120 (*p* < 0.001), demonstrating good discriminability for all items, which were therefore retained.

The second EFA yielded a KMO value of 0.943 and a Bartlett’s test of sphericity *χ*^2^ = 4175.680 (*p* < 0.001), indicating that the data were suitable for factor extraction. Using principal component analysis with varimax rotation, three factors with eigenvalues >1 were retained, accounting for 61.737% of the total variance. Guided by eigenvalues, the scree plot, communalities, interpretability, and cumulative variance, a 3-dimensional and 13-item OPABMS Scale was constructed. All item loadings exceeded 0.60 and no cross-loadings were observed. Details are shown in Table 1.

**Table 1:** OPABMS second exploratory factor analysis (*n* = 355).

Factor number	Factor name	Item number	Factor loading	Communality	Eigenvalue	Variance (%)	Cumulative variance (%)
F1	AB				2.905	22.349	22.349
		A1	0.775	0.633			
		A2	0.711	0.626			
		A3	0.678	0.660			
		A4	0.625	0.625			
F2	PB				2.761	21.242	43.591
		P1	0.712	0.536			
		P2	0.706	0.616			
		P3	0.678	0.586			
		P4	0.650	0.625			
		P5	0.632	0.513			
F3	CB				2.359	18.146	61.737
		C1	0.826	0.769			
		C2	0.786	0.697			
		C3	0.734	0.684			
		C4	0.630	0.532			

The specific items of the OPABMS Scale are as follows: (a) Avoidance Behavior (AB) refers to older patients’ perception of medical staff’s conduct aimed at evading contact with them. It includes four items: (A1) When I asked about the cause of my illness, the medical staff did not give me a detailed explanation because of my old age. (A2) When providing medical services to me, the medical staff showed an indifferent expression because of my old age. (A3) When I asked about medical treatment matters, the medical staff ignored me because of my old age. (A4) When I needed medical services, I had to call them multiple times before they came because of my old age. (b) Perfunctory Behavior (PB) refers to older patients’ perception of medical staff’s attitudes and behaviors characterized by irresponsibility and superficial engagement. It consists of five items: (P1) Because of my old age, the medical staff prescribed medications or ordered tests without standard diagnostic procedures, such as inspection, auscultation and olfaction, inquiry, and pulse-taking (traditional Chinese medicine) or inspection, palpation, percussion, and auscultation (Western medicine). (P2) Because of my old age, the medical staff prescribed medications that were unhelpful for my illness. (P3) Because of my old age, the medical staff ordered numerous tests without discussing them with me. (P4) Because of my old age, the medical staff ordered tests that were unhelpful for my illness. (P5) Because of my old age, the medical staff prescribed medication or ordered tests directly before I finished describing my symptoms. (c) Complaint Behavior (CB) refers to older patients’ perception of medical staff’s expressions of dissatisfaction toward them. It includes four items: (C1) When I followed up on medical treatment matters, the medical staff reproached me, saying “Why do you keep asking over and over again?” because of my old age. (C2) When I followed up on medical treatment matters, the medical staff yelled at me loudly because of my old age. (C3) When we disagreed, the medical staff said “Go ahead and file a complaint” because of my age. (C4) The medical staff spoke to me with a harsh tone because of my age.

#### 3.1.2 Confirmatory factor analysis of the OPABMS scale

CFA showed: *χ*^2^/df = 2.328, GFI = 0.939, AGFI = 0.911, NFI = 0.923, CFI = 0.954, RMR = 0.042, RMSEA = 0.061, all meeting the recommended thresholds and indicating good model fit. Thus, the 3-dimensional and 13-item structure of the OPABMS Scale is an appropriate model with satisfactory construct validity. Detailed figures are presented in Table 2.

**Table 2:** Comparison of model fit indices (*n* = 355).

Index	*χ*^2^/df	GFI	AGFI	NFI	CFI	RMR	RMSEA
Fit Criterion	<3	>0.9	>0.9	>0.9	>0.9	<0.05	<0.08
Revised Model	2.328	0.939	0.911	0.923	0.954	0.042	0.061

Convergent validity test showed that the AVE values were 0.467 for AB, 0.438 for PB, and 0.568 for CB, with corresponding CR of 0.776, 0.793, and 0.840. Such results may be due to the relatively low factor loadings of some items. Fornell and Larcker (1981) proposed that convergent validity remains adequate when CR > 0.60 even if AVE < 0.50, which is widely supported (Avilés-Noles et al., 2025; Huang et al., 2013). Lam even considered an AVE above 0.40 acceptable when CR is adequate (Lam, 2012). Furthermore, Moral de la Rubia (2019) argued that AVE > 0.50 criterion can be difficult to meet and may obscure the evaluation of actual convergent validity. He advocated for flexible criteria considering item count, factor loadings, and reliability. For instance, assuming equal standardized factor loadings and a reliability of 0.70, an AVE of at least 0.368 (for 4 items) or 0.318 (for 5 items) is sufficient. The indicators in this study meet these more flexible standards. Therefore, despite the marginally low AVE for AB and PB, the strong CR values support sufficient convergent validity. Discriminant validity was confirmed, as all HTMT values were below the conservative threshold of 0.85 (Pawellek et al., 2026): AB-PB = 0.75, AB-CB = 0.75, and PB-CB = 0.83.

#### 3.1.3 Criterion validity

The Pearson correlation coefficients between the overall and each dimension of the OPABMS and the patient satisfaction scale were −0.392, −0.360, −0.352 and −0.308, respectively, with *p* < 0.001. According to Cohen (1988), correlation coefficients (*r*) of 0.30 represent medium effects, supporting the criterion validity of the OPABMS.

#### 3.1.4 Internal consistency of the OPABMS scale

The overall Cronbach’s Alpha value of the OPABMS Scale was 0.899; Cronbach’s Alpha value of each dimension ranged from 0.779 to 0.801, and the split-half reliability was 0.922, indicating good reliability.

#### 3.1.5 Measurement invariance

M1, M2, and M3 were used to test measurement invariance across usual residence, while M1, M2, M3, and M4 were used to test measurement invariance across gender. All models demonstrate satisfactory outcomes. These results suggest equivalent construct measurement across usual residence and gender. Detailed figures are presented in Table 3.

**Table 3:** Analysis of measurement invariance (*n* = 710).

Model	*χ* ^2^	df	CFI	TLI	RMSEA	SRMR	∆CFI	∆RMSEA
Measurement invariance across usual residence								
M1: configural invariance	409.637	124.000	0.926	0.907	0.081	0.047		
M2: metric invariance	431.027	134.000	0.923	0.910	0.079	0.054	0.003	0.002
M3: scalar invariance	455.173	144.000	0.919	0.913	0.078	0.056	0.004	0.001
Measurement invariance across gender								
M1: configural invariance	410.649	124.000	0.927	0.908	0.081	0.047		
M2: metric invariance	435.774	134.000	0.923	0.911	0.080	0.054	0.004	0.001
M3: scalar invariance	449.611	144.000	0.922	0.916	0.077	0.054	0.001	0.003
M4: strict invariance	488.104	157.000	0.916	0.916	0.077	0.059	0.006	0.000

#### 3.1.6 Common method biases test

As all data were collected via self-report questionnaires, we assessed the potential for common method bias using the ULMC technique. The results showed that after adding the method factor, the changes in CFI, TLI, RMSEA, and SRMR were all less than 0.05. These results indicate no substantial common method bias.

### 3.2 Sample characteristics and descriptive statistics

#### 3.2.1 Sample characteristics

A total of 710 older patients were surveyed. Among the participants, 266 (37.46%) were male and 444 (62.54%) were female; the mean age was 69.36 years (*SD* = 6.74). 373 (52.54%) participants resided in urban areas, and 516 (72.68%) were married or cohabiting. 323 (45.49%) were farmers, which was the largest occupational group. 321 (45.21%) participants had completed primary school or less. When seeking routine medical care, 545 individuals (76.76%) were usually accompanied by family members. 283 (39.86%) reported a monthly per-capita household income of <3,000. Detailed figures are presented in Table 4.

**Table 4:** Sample characteristics and univariate analysis of OPABMS (*n* = 710).

Variables	*n* (%)	OPABMS M(SD)	AB M(SD)	PB M(SD)	CB M(SD)
Gender					
Male	266 (37.46%)	2.13 (0.70)	2.27 (0.84)	2.22 (0.76)	1.87 (0.79)
Female	444 (62.54%)	2.05 (0.62)	2.21 (0.69)	2.11 (0.75)	1.82 (0.71)
*t*		0.871	1.404	0.856	1.825
Age					
60–69	331 (46.62%)	2.08 (0.69)	2.20 (0.74)	2.17 (0.81)	1.84 (0.78)
70–79	300 (42.25%)	2.05 (0.59)	2.22(0.74)	2.10 (0.68)	1.81 (0.67)
≥80	79 (11.13%)	2.21 (0.68)	2.43 (0.79)	2.24 (0.79)	1.96 (0.85)
*F*		1.977	3.180*	1.248	1.214
Usual residence					
Urban	373 (52.54%)	2.00 (0.59)	2.13 (0.67)	2.07 (0.71)	1.79 (0.68)
Rural	337 (47.46%)	2.16 (0.71)	2.34 (0.81)	2.24 (0.80)	1.90 (0.80)
*t*		−3.280**	−3.655**	−2.867**	−1.946
Marital status					
Married/cohabiting	516 (72.68%)	2.07 (0.65)	2.21 (0.73)	2.14 (0.76)	1.84 (0.75)
Single (never married/divorced/widowed)	194 (27.32%)	2.11 (0.67)	2.29 (0.79)	2.17 (0.75)	1.85 (0.72)
*t*		−0.660	−1.285	−0.358	−0.133
Occupation					
Farmer	323 (45.49%)	2.14 (0.67)	2.34 (0.78)	2.18 (0.78)	1.90 (0.77)
Government/public institution	115 (16.20%)	2.03 (0.66)	2.11 (0.72)	2.11 (0.82)	1.86 (0.73)
Enterprise employee	145 (20.42%)	2.06 (0.64)	2.18 (0.71)	2.15 (0.72)	1.83 (0.74)
Self-employed	51 (7.18%)	1.92 (0.69)	2.05 (0.76)	2.05 (0.72)	1.63 (0.77)
Other	76 (10.70%)	2.03 (0.56)	2.18 (0.69)	2.16 (0.68)	1.73 (0.57)
*F*		1.714	3.429**	0.394	2.018
Education					
Primary or below	321 (45.21%)	2.15 (0.70)	2.32 (0.80)	2.23 (0.80)	1.89 (0.79)
Junior high	184 (25.92%)	2.03 (0.64)	2.22 (0.75)	2.05 (0.73)	1.80 (0.71)
Senior high/technical secondary	142 (20.00%)	2.06 (0.60)	2.13 (0.60)	2.17 (0.72)	1.85 (0.73)
College or above	63 (8.87%)	1.92 (0.51)	2.04 (0.71)	2.01 (0.65)	1.69 (0.58)
*F*		3.178**	3.976**	2.938*	1.624
Accompaniment at routine visits					
Yes	545 (76.76%)	2.07 (0.64)	2.22 (0.73)	2.13 (0.75)	1.84 (0.74)
No	165 (23.24%)	2.11 (0.70)	2.27 (0.81)	2.21 (0.79)	1.83 (0.75)
*t*		−0.764	−0.760	−1.219	0.140
Monthly per-capita household income					
<3,000	283 (39.86%)	2.18 (0.72)	2.33 (0.79)	2.25 (0.81)	1.94 (0.82)
3,000–5,999	271 (38.17%)	2.02 (0.59)	2.18 (0.71)	2.08 (0.71)	1.78 (0.68)
≥6,000	156 (21.97%)	2.02 (0.59)	2.14 (0.71)	2.10 (0.73)	1.78 (0.67)
*F*		5.217**	4.343*	3.818*	3.871*

*Refers to *p* < 0.05; **refers to *p* < 0.01; ***refers to *p* < 0.001.

#### 3.2.2 Univariate analysis of OPABMS

The overall mean score for OPABMS differed significantly across place of usual residence, educational level, and monthly per-capita household income (*p* < 0.01). At the subscale level, AB scores varied significantly across age, usual residence, occupation, educational level, and monthly per-capita income (*p* < 0.05). Variations in PB scores were associated with usual residence, educational level, and monthly per-capita income (*p* < 0.05). CB scores differed only by monthly per-capita income (*p* < 0.05). Full details are presented in Table 4.

#### 3.2.3 Descriptive statistics and correlation analysis

The mean item score of OPABMS was 2.08 (*SD* = 0.65) and the sub-scale means ranked from highest to lowest were AB, PB, and CB, all lying slightly below the scale midpoint. The corresponding mean item score of OPL was 3.45 (*SD* = 0.77), indicating a moderately high level of loyalty. Bivariate correlations revealed significant inverse associations between OPABMS (total and all three sub-scales) and OPL (total and each dimension) (*p* < 0.001). Full details are displayed in Table 5.

**Table 5:** Summary table of descriptive statistics and correlation analysis (*n* = 710).

Variables	*M (SD)*	OPABMS	AB	PB	CB	OPL	I-REC	I-REV	I-PWM
OPABMS	2.08 (0.65)	1.000							
AB	2.23 (0.75)	0.846***	1.000						
PB	2.15 (0.76)	0.898***	0.625***	1.000					
CB	1.84 (0.74)	0.860***	0.611***	0.659***	1.000				
OPL	3.45 (0.77)	−0.390***	−0.370***	−0.347***	−0.300***	1.000			
I-REC	3.47 (0.85)	−0.341***	−0.329***	−0.286***	−0.276***	0.865***	1.000		
I-REV	3.52 (0.91)	−0.303***	−0.270***	−0.278***	−0.240***	0.859***	0.632***	1.000	
I-PWM	3.36 (0.91)	−0.363***	−0.356***	−0.330***	−0.257***	0.855***	0.617***	0.579***	1.000

*Refers to *p* < 0.05; **refers to *p* < 0.01; ***refers to *p* < 0.001.

### 3.3 Association between OPABMS and OPL

Collinearity diagnostics indicated no multicollinearity in the multivariable models, with all VIF < 5 and tolerance values >0.20, which are well within acceptable thresholds. Two-step hierarchical models were constructed to examine the association between OPABMS and OPL. Each loyalty dimension served as the dependent variable. Model 1 entered demographic covariates (gender, age, usual residence, etc.); Model 2 added the three OPABMS sub-scales. After covariate control, AB significantly and negatively correlated with I-REC, accounting for 11.6% of its variance. AB and PB together showed significant negative associations with both I-REV (8.4% of variance explained) and I-PWM (14.1% of variance explained). Detailed coefficients are reported in Table 6.

**Table 6:** Hierarchical regression analysis of the association between OPABMS and OPL (*n* = 710).

Variables	I-REC	I-REV			I-PWM	
	Model 1	Model 2	Model	Model 2	Model 1	Model 2
Demographic covariates						
Gender	0.043	0.026	0.070	0.053	0.038	0.017
Age	0.004	0.011	0.017	0.020	−0.022	−0.015
Usual residence	0.014	0.040	0.036	0.061	0.062	0.100*
Marital status	−0.039	−0.035	0.005	0.008	−0.033	−0.028
Occupation	−0.006	−0.017	−0.045	−0.050	0.019	0.017
Education	0.128**	0.111*	0.122*	0.110	0.097*	0.077
Accompaniment at routine visits	−0.092*	−0.085*	−0.158***	−0.151***	−0.100*	−0.087*
Monthly per-capita household income	−0.045	−0.054	0.063	0.054	−0.004	−0.011
OPABMS						
AB		−0.212***		−0.134**		−0.247***
PB		−0.090		−0.135**		−0.185***
CB		−0.089		−0.066		0.015
*R^2^*	0.022	0.138	0.052	0.136	0.022	0.162
Δ*R*		0.116***		0.084***		0.141***
*F*	2.010*	10.153***	4.853***	10.001***	1.928	12.283***

*Refers to *p* < 0.05; **refers to *p* < 0.01; ***refers to *p* < 0.001.

## 4 Discussion

### 4.1 The OPABMS scale demonstrates satisfactory reliability and validity

This study shows that, in terms of reliability, the overall Cronbach’s Alpha value of the scale was 0.899, the Cronbach’s Alpha value of the three sub-dimensions ranged from 0.779 to 0.828, and the split-half reliability coefficient was 0.922. These values indicate satisfactory internal consistency for the OPABMS Scale. Regarding validity, construct validity was established through multiple analyses. Exploratory factor analysis extracted three common factors explaining 61.737% of the total variance; all item loadings exceeded 0.60 with no cross-loadings. Confirmatory factor analysis further demonstrated adequate model fit with all indices within acceptable ranges, supporting sound structural validity. Convergent validity and discriminant validity were also adequately established according to commonly accepted criteria. For criterion validity, this study selected older patients’ satisfaction as the criterion variable. Results showed that the total score and all dimensions of OPABMS were negatively correlated with older patients’ satisfaction (*r* = −0.308 to −0.392, *p* < 0.01), indicating adequate criterion validity. Furthermore, measurement invariance was confirmed across usual residence and gender, supporting the comparability of scores across subgroups. Therefore, the OPABMS Scale, comprising the three dimensions of AB, PB and CB, exhibits satisfactory reliability and validity, and can serve as an appropriate instrument for measuring OPABMS.

### 4.2 The current situation of OPABMS

Older patients in this study reported a slightly-below-moderate level of perceived ageist behaviors from medical staff, implying that the majority of medical institutions interact with older patients in a respectful and caring manner. Nevertheless, a non-trivial proportion of staff were perceived to display differential or unfair treatment. Among the three sub-scales, AB obtained the highest mean score. Two mechanisms may account for this pattern. First, medical staff may hold stereotypical views that older patients are difficult to understand or to cooperate with, and to reduce the hassle of interacting with them, they chose to avoid direct contact. Second, heavy workloads leave staff with insufficient time or emotional resources for in-depth communication, leading older patients to perceive avoidance from medical staff. Consequently, health authorities and medical institutions need to keep OPABMS on the agenda and particularly target AB.

### 4.3 Significant between-group differences in OPABMS

Significant between-group differences emerged. Patients aged 80 and above reported higher levels of OPABMS than those aged 60–69 and 70–79. A possible explanation is that progressive physiological decline after the eighth decade erodes self-worth (Sharma nath and Patra, 2024); simultaneously, older adults are more susceptible to social influence and highly sensitive to others’ attitudes (Su et al., 2024). Consequently, they are more likely to interpret ambiguous staff behaviors as discriminatory, resulting in elevated OPABMS scores. Rural residents reported higher scores than urban residents. This disparity may be rooted in China’s urban–rural dual structure, which is characterized by marked inequalities in the distribution of social-security coverage (Liu and Chu, 2024), health-care resources (Xie et al., 2021) and household income (Zhang, 2022). These structural inequities shape the subjective healthcare experiences of rural residents, increasing their vulnerability to perceiving ageist behaviors during clinical encounters. Farmers reported higher levels than other occupational groups; medical staff may harbour negative stereotypes about this population and attempt to limit contact, thereby amplifying perceived avoidance. Compared with older patients with higher education levels, older patients with lower education levels perceive higher levels of ageist behavior. This may be because older patients with lower education have insufficient theoretical knowledge and limited cognition, which prevents them from scientifically and comprehensively coping with various problems brought about by the aging process. At the same time, their social support is lower and their social resources are relatively scarce, making it difficult for them to obtain high-quality medical services (Qin et al., 2025), so that they perceive more ageist behaviors from medical staff. Finally, respondents in the lowest income category reported more ageist behaviors than their wealthier peers; constrained purchasing power reduces choice of provider (Cabañero-Garcia et al., 2025) and may expose poorer patients to lower-quality encounters. Collectively, these findings indicate that extra attention should be paid to very old, rural, farming, less educated and lower-income older patients. Interventions that improve communication skills, reduce time pressure and explicitly counter stereotypes among medical staff are likely to diminish perceived ageist behaviors in these high-risk sub-groups.

### 4.4 Association between OPABMS and OPL

OPABMS’s total score and all three sub-scales were inversely associated with OPL total score and three sub-scales (*p* < 0.001). Hierarchical regression showed that AB was associated with lower scores on I-REC, I-REV and I-PWM, whereas PB showed an additional negative association with I-REV and I-PWM. Collectively, OPABMS is negatively associated with OPL.

Previous work indicates that patients infer service quality from their healthcare experience (Zarei et al., 2014) and the quality of medical services is one of the determinants of patient loyalty (Zhou et al., 2017). Therefore, as a negative healthcare experience, OPABMS is associated with patients forming a negative evaluation of medical service quality and forgoing future use of the original service, which may correspond to lower patient loyalty. As older adults are high-frequency users of health services, their continued allegiance is central to organizational sustainability and competitive positioning. Policy makers and hospital managers should therefore embed non-technical care metrics into routine governance. Specific actions include: First, refine internal administrative protocols within medical institutions to comprehensively and effectively reduce OPABMS through multiple channels in daily management. Second, strengthen medical staff’s self-regulation to prevent ageist behaviors at their root, which may contribute to improving geriatric service quality and advancing age-friendly medical institution construction.

### 4.5 Implications and limitations

In summary, the scale developed in this study can serve as a valid instrument for measuring OPABMS. The overall level of OPABMS was found to be moderately low. These behaviors, especially AB and PB, were significantly associated with lower loyalty across all three dimensions (I-REC, I-REV, and I-PWM). Health authorities, hospital administrators, and medical staff should consider OPABMS as a potential quality indicator. Embedding age-inclusive communication standards in internal regulations and strengthening medical staff’s self-regulation may help reduce OPABMS, improve older patients’ healthcare experience and ultimately support OPL.

Limitations should be acknowledged. (a) This study was constrained by its sampling frame. The use of convenience sampling may have introduced selection bias and reduced sample representativeness. Future research should employ probability sampling methods, such as stratified or cluster sampling, combined with multi-center collaboration, to enhance sample representativeness and the generalizability of findings. (b) Although conducting the survey outside clinical settings helped avoid courtesy bias, it may have introduced recall bias. Additionally, the 12-month recall period may further contribute to recall inaccuracies, which may compromise the accuracy of older patients’ reported perceptions. (c) As all information in this study was derived from the subjective reports of older patients, there may be self-report bias. (d) The cross-sectional design precludes causal inferences. (e) This study was conducted exclusively among older patients in China, and the findings may therefore possess cultural specificity, limiting their generalizability to other populations or contexts. (f) As an exploratory study, this study focused on scale development and initial validation. While it identified the structure of OPABMS and its association with OPL, it did not examine underlying mediating or moderating mechanisms. Future research should incorporate additional intervention factors related to OPABMS and test their mediating or moderating effects, thereby enabling the design of more targeted interventions to reduce such perceptions and providing administrators with more valuable evidence for integrating a positive view of ageing into the high-quality development of medical institutions.

## Data Availability

The raw data supporting the conclusions of this article will be made available by the authors, without undue reservation.
